# Correspondence: Flawed assumptions compromise water yield assessment

**DOI:** 10.1038/ncomms14795

**Published:** 2017-05-17

**Authors:** Lukas Gudmundsson, Peter Greve, Sonia I. Seneviratne

**Affiliations:** 1 Institute for Atmospheric and Climate Science, Department of Environmental Systems Science, ETH Zurich, Universitaetstrasse 16, Zurich 8092, Switzerland

*Nature Communications* 8:14795 doi: 10.1038/ncomms14795 (2017); Published 17 May 2017

In their recent study, Zhou *et al*. (hereafter Z15) aim at mapping global patterns of the relative contributions of climate and land-cover change on the water yield coefficient. However, implicit and not discussed assumptions on ‘typical changes' of the considered forcing factors^2^,^3^ raise questions on the physical integrity of substantial aspects of their analysis^4^. In fact, we show here that central findings of Z15 are reversed if physically and mathematically more justified assumptions are accounted for.

Z15 analyse partial differentials of a rearranged version of Fu's equations^5^,^6^ that predicts the long-term mean ratio of precipitation (*P*) and runoff (*R*), which they refer to as the water yield coefficient, as


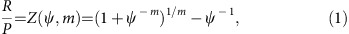


where the wetness index 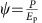 is defined as the long-term mean ratio of precipitation and potential evapotranspiration (*E*_p_). The parameter *m* is generally unknown and accounts for all other factors that might influence the partitioning of precipitation into runoff and evapotranspiration, including (but not limited to) the effect of land-cover change. Z15 subsequently compare 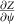, the partial derivative of equation (1) with respect to *ψ*, to 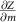, the partial derivative with respect to *m*, to determine whether variations of *ψ* or *m* have a greater influence on the water yield coefficient. More precisely, they define the relative contribution of *m* to changes in the water yield coefficient as


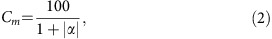


where 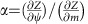. Figure 1a is equivalent to Fig. 6b presented by Z15 and shows the relative contribution of changes in *m* to changes in the water yield coefficient for different values of *ψ* and *m*. Figure 1b shows the resulting global patterns of the relative contribution of *m* to changes in the water yield coefficient and is qualitatively comparable to Fig. 7a of Z15. (Note that the figures could not be made identical as some of the global data considered by Z15 are no longer available online; instead alternative well established data^7^ were considered.) These figures suggest that changes in the water yield coefficient are less sensitive to changes in *m* in arid climates (*ψ*<1), which is inconsistent with alternative assessments^4^ and contradicts central statements made in the abstract of Z15.

Furthermore, conclusions based on the approach of Z15 are sensitive to rearrangements of equation (1)^4^. For example, equation (1) can be expressed as a function of the inverse wetness index, 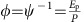, such that


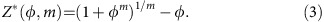


Following Z15 the relative contribution of *m* to changes in the water yield coefficient would read as


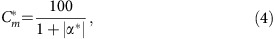


where 
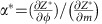
 (see Methods for partial derivatives). Figure 1c,d shows 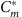 for different values of *m* and *φ*=*ψ*^−1^ as well as the resulting spatial pattern, which differ substantially from the original assessment (Fig. 1a,b). As a consequence, assessing *C*_*m*_ and 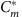 results in different conclusions, highlighting the physical inconsistency of the approach suggested by Z15.

To understand this issue it is necessary to recall that the respective contributions of changes in *ψ* and *m* on the water yield coefficient (or related variables) have to be analysed in the context of the total differential^4^,^8^. This implies that a change in the water yield coefficient (Δ*Z*) is related to changes in the wetness index (Δ*ψ*) and changes in all other factors (Δ*m*), such that


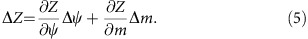


By comparing the partial derivatives directly, Z15 implicitly assume that Δ*ψ*=Δ*m*=1. Initially this appears a plausible assumption, as a unit change in both *ψ* and *m* is considered. It is however, important to note that a unit change in *ψ* does by no means have the same physical meaning as a unit change in *m*.

These issues can be resolved by making explicit assumptions on the magnitude of both Δ*ψ* and Δ*m*. The relative influence of the wetness index can then be re-written as


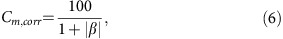


where 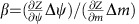. Although values of Δ*ψ* can for example be derived from observations or climate model projections of precipitation and potential evapotranspiration, this is not necessarily the case for *m*. We therefore employ a previously utilised approach^4^, which is not sensitive to rearrangements of Fu's equation and assess the respective influences of equal relative changes in both factors, such that 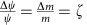, where *ζ* denotes the relative change. Figure 1e,f shows the relative contribution of changes in the wetness index to changes in the water yield coefficient under the assumption that both *ψ* and *m* have the same relative change. In contrast to the results of Z15 (Fig. 1a,b), the revised analysis (Fig. 1e,f) shows that the water yield coefficient is highly sensitive to changes in *m* in arid conditions and is consistent with previous assessments^4^.

In summary, central aspects of the analysis of Z15 are dependent on the strong—and not discussed—assumption that both *ψ* and *m* exhibit a unit change. However, a unit change in *ψ* has by no means the same physical interpretation as a unit change in *m* raising questions regarding the realism of their assessment. In addition, their approach is sensitive to simple rearrangements of the governing equation, highlighting issues with the physical and mathematical integrity of their approach. Both issues have significant effects on their analysis, which is illustrated by the fact that central results of the assessment are reversed if realistic assumptions on changes in the driving factors are accounted for. Consequently, the global maps of the relative contribution of *m* and *ψ* to changes in the water yield coefficient provided by Z15 are compromised, which implies that they cannot be used to assess the effects of climate or environmental change on the world's freshwater resources. Instead, alternative assessments that explicitly account for typical changes in the forcing factors (e.g. Fig. 1e,f) are required to derive reliable estimates of the relative contributions of the wetness index and other factors on the water yield coefficient.

## Methods

### Partial derivatives

The partial derivatives of equation (3) are


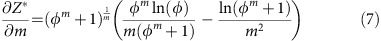


and


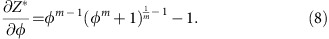


### Data availability

**Figure 1:**
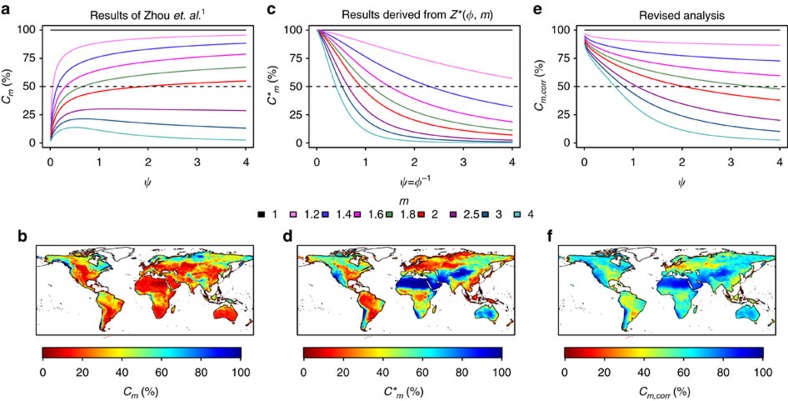
The relative contribution of *m* to changes in the water yield coefficient. (**a**, Theoretical illustration; **b**, global application) show inferred relative contribution of *m* to changes in the water yield coefficient using the *C*_*m*_ definition of Z15 (equation (2)). (**c**,**d**) show 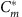 (equation (4)) which is derived using the approach of Z15 but from a rearranged version of the governing equation. The difference between *C*_*m*_ and 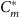 highlights issues with the integrity of the approach proposed by Z15, as both are aiming at quantifying the relative contribution of *m* on the water yield coefficient. Note that 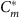 is computed using the inverse wetness index (*&φ;*=*ψ*^−1^) but is plotted against the wetness index in **c** to facilitate a visual comparison with the other panels. Note that **a**–**d** make implicit but physically not meaningful assumptions on changes in both the wetness index (*ψ*) and all other factors (*m*) as discussed in the text. (**e**,**f**) show *C*_*m*,*corr*_, a revised quantification of the relative contribution of *m* to changes in the water yield coefficient, that makes the physically meaningful assumption that both *m* and *ψ* do exhibit the same relative change (equation (6)). Note that the results are sensitive to this assumption, and will change, for example, if the change in *ψ* does not have the same relative change as *m*^4^. The global analysis is based on long-term mean precipitation, runoff and net radiation (*R*_*n*_) from the ERA-Interim/Land Data^7^, where net radiation was expressed in water equivalent units and acts as a first-order estimate of potential evapotranspiration (that is, *E*_*p*_=*R*_*n*_/*λ*, where *λ* is the latent heat of vaporisation). The parameter *m* was identified by fitting equation (1) to the above mentioned data at each grid-cell.

The ERA-Interim/Land Data^7^ that are underlying Fig. 1b,d,f are available through the European Centre for Medium-Range Weather Forecasting (ECMWF, http://www.ecmwf.int/en/research/climate-reanalysis/era-interim/land).

## Additional information

**How to cite this article:** Gudmundsson, L. *et al*. Correspondence: Flawed assumptions compromise water yield assessment. *Nat. Commun.* **8,** 14795 doi: 10.1038/ncomms14795 (2017).

**Publisher's note**: Springer Nature remains neutral with regard to jurisdictional claims in published maps and institutional affiliations.

## References

[b1] Zhou G. . Global pattern for the effect of climate and land cover on water yield. Nat. Commun. 6, 5918 (2015). 25574930 10.1038/ncomms6918

[b2] Berghuijs W. R. & Woods R. A. Correspondence: space-time asymmetry undermines water yield assessment. Nat. Commun. 7, 11603 (2016). 27176558 10.1038/ncomms11603 PMC4865832

[b3] Chen X., Wei X., Sun G., Zhou P. & Zhou G. Correspondence: reply to ‘space-time asymmetry undermines water yield assessment'. Nat. Commun. 7, 11604 (2016). 27176655 10.1038/ncomms11604 PMC4865858

[b4] Gudmundsson L., Greve P. & Seneviratne S. I. The sensitivity of water availability to changes in the aridity index and other factors—A probabilistic analysis in the Budyko-space. Geophys. Res. Lett 43, 6985–6994 (2016).

[b5] Zhang L. . A rational function approach for estimating mean annual evapotranspiration. Water Resour. Res. 40, W02502 (2004).

[b6] Fu B. On the calculation of the evaporation from land surface [in chinese]. Sci. Atmos. Sin. 1, 23–31 (1981).

[b7] Balsamo G. . Era-interim/land: a global land surface reanalysis data set. Hydrol. Earth Syst. Sci. 19, 389–407 (2015).

[b8] Roderick M. L. & Farquhar G. D. A simple framework for relating variations in runoff to variations in climatic conditions and catchment properties. Water Resour. Res. 47, W00G07 (2011).

